# A Rare Case of Horseshoe Kidney With Multiple Atrial Myxomas Presenting as Cerebrovascular Accident

**DOI:** 10.7759/cureus.26362

**Published:** 2022-06-27

**Authors:** Andrea Marin, Ankita Prasad, Sharon Hechter, Lwoodsky Charles, Priya Patel, Mehnoor Durrani, Ayesha Imtiaz, Nagapratap Ganta, Arthur Okere, Varun Vankeshwaram, Pramil Cheriyath

**Affiliations:** 1 Internal Medicine, Hackensack Meridian Health Ocean Medical Center, Brick, USA; 2 Internal Medicine, Hackensack Meridian health Ocean Medical Center, Brick, USA; 3 Cardiology, Hackensack Meridian Health Ocean Medical Center, Brick, USA; 4 Medicine, Hackensack Meridian Health Ocean Medical Center, Brick, USA

**Keywords:** carney syndrome, renal failure, stroke, embolize, horseshoe kidney, familial, multiple, atrial myxoma

## Abstract

Myxomas are benign tumors of mesenchymal origin, containing a few pluripotent cells in the myxomatous stroma. They usually present at 30-40 years of age and are more common in females than males. These tumors mostly arise in the atria and protrude into the atrial lumen. They cause constitutional symptoms like fever and weight loss and obstructive symptoms related to outflow obstruction in the heart. Some tumors are more fragile and cause embolism and may present as stroke. Mostly sporadic but familial cases and myxomas associated with Carney syndrome (CNC) tend to be multiple. Here, we report a case of a 40-year-old female with a stroke due to embolization from multiple myxomas. She had no family history of myxoma and had no skin findings or other tumors associated with CNC. She also had an atrophied horseshoe kidney with renal failure. The association of a horseshoe kidney with myxoma is rarely reported. In an extensive literature search, we could only find only one other case. Atrial myxomas were detected while investigating the cause of stroke. Our patient gradually improved and was advised surgical removal of the myxomas, which is the treatment of choice.

## Introduction

Myxomas have a reported incidence of 0.001% to 0.3% by autopsy [1]. Cardiac myxomas are benign cardiac tumors consisting of a few cells originating from multipotent mesenchyme cells in a mucopolysaccharide stroma. These cells produce vascular endothelial growth factors for tumor growth [2]. Myxomas are the most common primary cardiac tumors affecting adults and account for more than half of the benign primary cardiac tumors [3]. The clinical features associated with myxoma are either embolism, effect on cardiac hemodynamics by interfering with the opening and closure of cardiac valves, and constitutional symptoms. These tumors are primarily sporadic. Only a tiny percentage are familial. Familial cases tend to be multiple or associated with Carney syndrome (CNC), a rare genetic disorder with multiple tumors involving the thyroid, adrenal, parathyroid, and pituitary glands, testis, ovaries, and breast, schwannomas, cardiac myxomas, and pigmentation abnormalities around the face, neck, and trunk. Usually, multiple atrial myxomas are observed in CNC, and there is a tendency for recurrence after resection. These tumors arise from the atrial septum and grow toward the atrial cavity. About 80% of myxomas are in the left atrium; the remainder is in the right atrium. Smaller tumors are more friable and tend to embolize, most commonly in the brain. Cardiac myxoma can embolize up to 50% of patients [4]. The embolic complications of an atrial myxoma are due to fragments of the myxoma itself or due to thrombotic material covered with tumor cells [3]. Cerebral arteries are involved in at least half of the cases [4]. Atrial myxomas have been estimated to cause up to 0.5% of ischemic strokes [5]. Several reports have described small cerebral aneurysms secondary to myxoma emboli [6-8]. Here, we are investigating a case of a 40-year-old female with a cerebrovascular incident that led to the diagnosis of multiple left atrial myxomas. Incidentally, the patient has an atrophied horseshoe kidney with renal failure and is on hemodialysis.

## Case presentation

**Figure 1:**
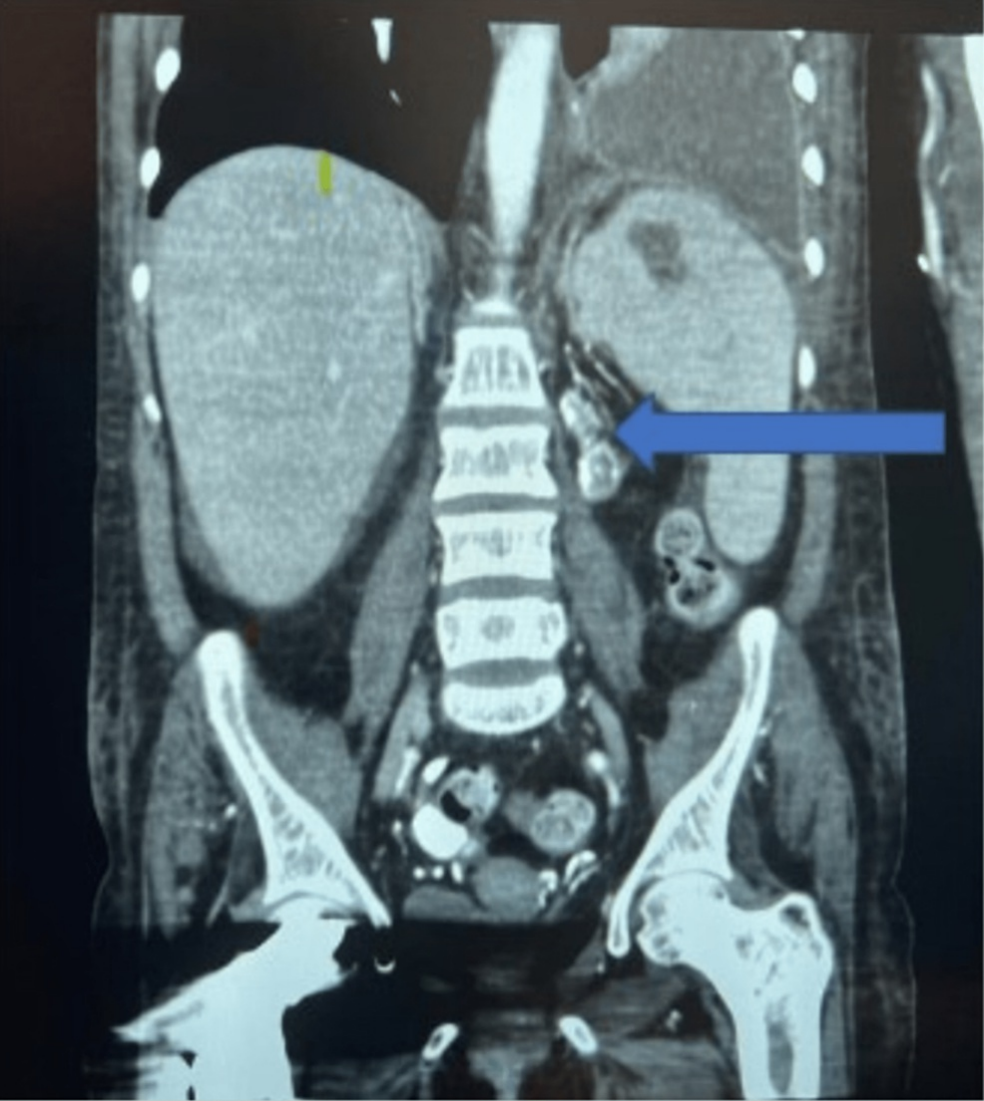
Abdomen imaging showing the atrophied horseshoe kidney (green arrow)

Our patient was a 40-year-old female with a history of end-stage renal disease (ESRD) on hemodialysis due to focal segmental glomerulosclerosis with horseshoe kidney (Figure 1), hypertension, and post parathyroidectomy.

**Figure 2:**
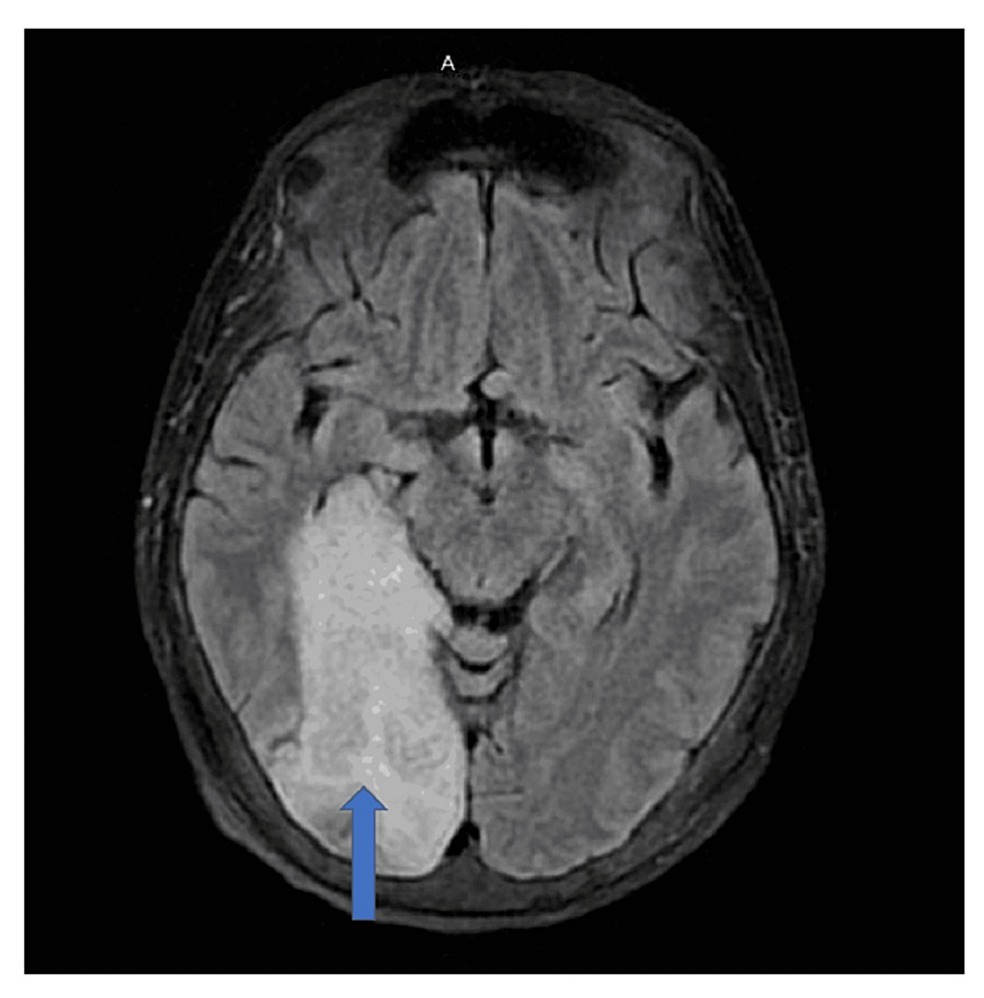
MRI brain showing posterior cerebral artery infarct (blue arrow)

She had a history of a recent change of hemodialysis access to the right jugular vein three days prior due to a non-functioning arteriovenous fistula (AVF). She presented with bilateral worsening lower limb weakness for 24 hours. She had no associated headache, loss of consciousness, cough, neck pain, back pain, trauma, or bowel/ bladder abnormalities. She has no previous history of similar episodes. She presented to the emergency room in shock, with hypotension, and was shifted to the intensive care unit after fluid resuscitation. Her vitals at presentation were temperature 97.2F, blood pressure 80/47 mm, and heart rate 106/minute. She continued to have persistent hypotension for many days, requiring pressor support. The new hemodialysis (HD) access had signs of infection. Blood cultures grew Staphylococcus aureus, for which she was given Vancomycin. The Perm A Cath was removed and replaced. Cultures obtained from the HD catheter also grew Staphylococcus aureus. Further evaluation of the suspected stroke showed a right posterior cerebral artery acute infarct (Figure 2).

**Figure 3:**
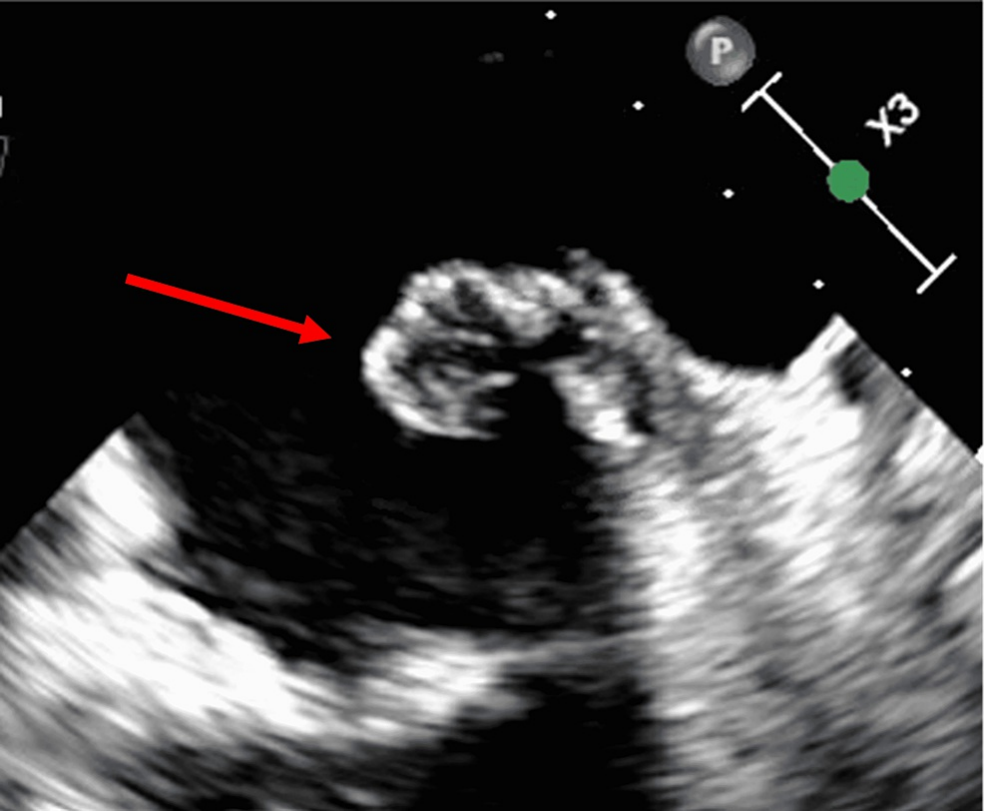
Transesophageal echocardiography (TEE) showing the largest myxoma, 2 cm in size (red arrow)

**Figure 4:**
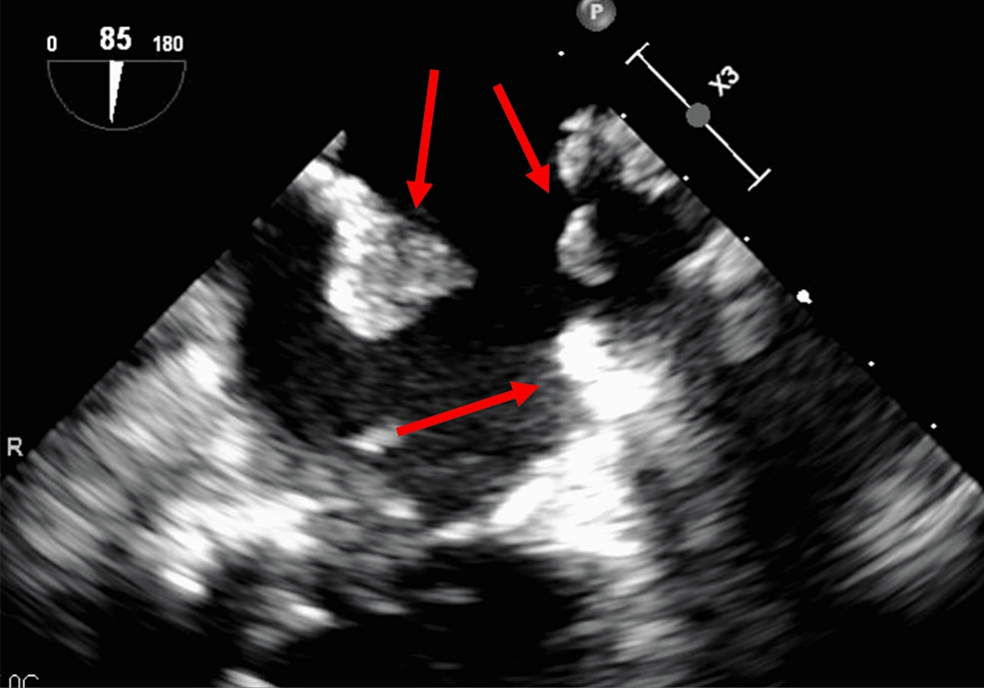
Transesophageal echocardiography (TEE) showing multiple atrial myxomas (red arrows)

The patient had resultant left upper and lower extremity weakness with left hemianopsia. A stroke workup showed the presence of multiple left atrial myxomas on a transesophageal echocardiogram (TEE). Three pedunculated myxomas were noted, and the largest was greater than 2 cm in size (Figures 3-4).

A cardiothoracic evaluation was done to diagnose the atrial mass and investigate a possible biopsy. Cardiac MRI was done, which showed multiple masses, including a mass in the left ventricle. The patient declined the biopsy. She did not have a family history of cardiac myxomas. She previously did not have an echocardiogram done before this event. She did not have pigmented skin or mucosal lesions. The patient’s endocrine history involved secondary hyperparathyroidism, but she did not have any other endocrinological blood tests; no other tumors were found after extensive workup so we ruled out CNC.

She was evaluated by cardiac surgery for myxoma. Medical management for four weeks followed by repeat echocardiography was recommended. Repeat MRI brain showed encephalomalacia in the right parietal lobe with new cortical hyper attenuation, laminar necrosis, and encephalomalacia in the left cerebellum. A new left upper extremity arteriovenous fistula (AVF) was created for hemodialysis. She completed antibiotics from the last positive culture evaluation and was discharged to follow up for atrial myxoma surgery, stroke rehabilitation, and end-stage renal disease management.

## Discussion

Myxomas account for more than half of all benign cardiac tumors, followed by fibroelastosis and lipoma. These are more common in females, and fibroelastosis is more common in males [1]. About 7% of myxoma cases may be familial, especially in CNC associated with cutaneous pigmentation, endocrine abnormalities, and multiple tumors [2]. Our patient had multiple left atrial myxomas but no family history of myxoma or related complications. She did not have any skin manifestations of CNC. On doing an extensive literature search using the keywords ‘horseshoe kidney’ and ‘atrial myxoma' in the PubMed, Google Scholar, and Cochrane databases, we could only find one case report of atrial myxoma with horseshoe kidney. It was present with CNC and was reported by Conroy S et al. [9].

Stroke may be a presentation for many left atrial myxomas. In 2019, a systemic review and meta-analysis done by Liu Y et al. identified New York Heart Association (NYHA) class I/II, hypertension, irregular tumor surface, atypical tumor location, the narrow base of the tumor, and increased fibrinogen as significant embolism risk factors in myxoma patients [10]. Most embolization happens to the spleen, adrenals, intestine, kidneys, abdominal aorta, coronary arteries, mesentery, and limb arteries [11].

Cardiac myxomas can interfere with the closure of heart valves, causing mitral valve regurgitation, dyspnea, cough, edema, and fatigue. A low-pitched diastolic sound known as tumor plop is heard on cardiac examination in about 15% of the patients [12]. Presenting symptoms for most cardiac myxomas are dizziness, palpitations, dyspnea, and congestive heart failure [13]. Myxomas can also cause syncope and sudden cardiac death [13]. They cause constitutional symptoms like weight loss and fatigue with laboratory abnormalities (anemia, increased globulins, increased erythrocyte sedimentation rate (ESR), and increased C-reactive protein (CRP). In a clinical study done by Keeling IM et al. on 49 cardiac myxoma patients who were followed for 24 years, cardiac signs appeared in up to 94% of the patients, and constitutive symptoms due to immunologic alterations presented in up to 87.5% [4]

TTE has been considered the procedure of choice for diagnosing and assessing atrial myxoma [14]. TEE can provide high-quality images of the heart and great arteries, allowing clear visualization of structures difficult to detect by TTE [14]. Cardiac MRI and computed tomography (CT) can provide additional information before planning resection, which should be done early to prevent complications. However, recurrence has been seen occasionally after resection, especially in cases of multiple myxomas that are familial or associated with CNC [15].

## Conclusions

Non-syndromic cases of multiple atrial myxomas with horseshoe kidneys have not been reported in the medical literature. The first case of myxoma with horseshoe kidneys was regarding a CNC patient. This happens to be the first case report of a patient with both features but no CNC. It is worth studying the presence of renal anomalies associated with myxomas to get a better insight into the genes involved, whether it is a one-of-a-kind case or if there are any syndromes associated with myxoma. If such an association exists, gene identification might be helpful to diagnose the presence of myxoma early in patients with horseshoe kidneys and prevent embolic complications.
